# A fast validation test of gene regulatory network models via the Fokker-Planck equation

**DOI:** 10.1007/s10867-025-09681-x

**Published:** 2025-05-19

**Authors:** Natalia López-Paleta, Eduardo Moreno-Barbosa, Jorge Velázquez-Castro

**Affiliations:** Facultad de Ciencias Físico Matemáticas, Benemérita Universidad Autónoma de Puebla, Puebla, 72570 Puebla México

**Keywords:** Gene regulatory network, Dynamical model, Fokker-Planck equation, Microarray

## Abstract

Since Waddington proposed the concept of the “epigenetic landscape” in 1957, researchers have developed various methodologies to represent it in diverse processes. Studying the epigenetic landscape provides valuable qualitative information regarding cell development and the stability of phenotypic and morphogenetic patterns. Although Waddington’s original idea was a visual metaphor, a contemporary perspective relates it to the landscape formed by the basins of attraction of a dynamical system describing the temporal evolution of protein concentrations driven by a gene regulatory network. Transitions among these attractors can be driven by stochastic perturbations, with the cell state more likely to transition to the nearest attractor or to the one that presents the path of least resistance. In this study, we define the epigenetic landscape using the free energy potential obtained from the solution of the Fokker-Planck equation on the regulatory network. Specifically, we obtained a numerical approximate solution of the Fokker-Planck equation describing the *Arabidopsis thaliana* flower morphogenesis process. We observed good agreement between the coexpression matrix obtained from the Fokker-Planck equation and the experimental coexpression matrix. This paper proposes a method for obtaining this landscape by solving the Fokker-Planck equation (FPE) associated with a dynamical system describing the temporal evolution of protein concentrations involved in the process of interest. As these systems are high-dimensional and analytical solutions are often unfeasible, we propose a gamma mixture model to solve the FPE, transforming this problem into an optimization problem. This methodology can enhance the analysis of gene regulatory networks by directly relating theoretical mathematical models with experimental observations of coexpression matrices, thus providing a discriminating technique for competing models.

## Introduction

In recent years, dynamic systems theory has been able to make inferences about the phenotypic characteristics of some species from gene regulatory models [1–7]. Establishing a relationship between these phenotypic characteristics and genetic expression will enable the prediction of phenotypic effects due to changes in gene network reconfiguration or alterations in expression factors. Thus, establishing this relationship is a fundamental problem of systems biology and will facilitate the design and evaluation of particular and targeted therapies based on control over certain expression factors. To achieve this goal, a first problem is determining the regulatory network created by the interaction between the different genes and proteins in the cell and its dynamics.

Knowledge of the structure of a particular genetic regulation network (GRN) allows the determination of the state of health and function of cells. Therefore, it can be used as a diagnostic tool. Several methods have been developed to infer GRN from gene expression data [8–18]. In the last few years, also several machine learning techniques have been developed [19–23] to infer GNRs. Each methodology can provide different results owing to the mathematical assumptions used to reconstruct the interaction network from the data. Thus, this is an active area of research, and there is no consensus on a standard methodology for inferring GRNs [24]. Its even known that most methods provide an approximate picture of the underlying network [24]. Despite these shortcomings, there is a large body of knowledge of GRNs for different systems. Thus, it is convenient to evaluate the predictions of a network and compare them with the observed data. Gene expression databases [25, 26] are currently used to identify correlations between gene activation. Conversely, the properties and behavior of dynamical gene regulatory networks have been studied using theoretical models [27–31]. Two types of dynamic models are most frequently used: Boolean and continuous models. Boolean models represent the system state, with 1 s and 0 s representing the activation or non-activation of each gene involved. The discrete-time evolution rules determine the dynamics. Continuous models provide a framework for describing the temporal dynamics of gene product concentrations in a continuous-time context. These models are underpinned by the principles governing reaction rates among various biological components. We propose that the contemporary interpretation of the epigenetic landscape serves as a critical nexus connecting empirical gene expression data with theoretical dynamical models of gene regulatory networks (GRNs). This integration may enhance our comprehension of the intricate regulatory mechanisms governing gene expression and its broader biological implications.

Morphogenetic evolution follows from the complex interactions between genes in the network and was represented by C.H. Waddington in 1957 for what he termed the epigenetic landscape [32]. Waddington proposed that the morphogenesis process can be represented metaphorically as a ball rolling downhill through a landscape of mountains and valleys. Currently, efforts are being made to formalize the concept of the epigenetic landscape as an analogy of Lyapunov functions or energy potentials. The dynamic system attractors are at the bottom of the basins, and the steady state in each attractor characterizes each type of cell or its phenotypic condition [33].

These systems have as many dimensions as genes involved in the network; therefore, for realistic systems, the number of dimensions is high. The high dimensionality of these systems has presented challenges in analyzing this type of phenomenon and deducing the epigenetic landscape [34]. Another challenge is the inherently stochastic nature of these systems, which necessitates the development of efficient techniques for finding solutions. Recently, a methodology was proposed to tackle this problem effectively [35, 36], achieving good results. This work aims to find a stationary probability distribution of concentrations, thus complementing the previously mentioned studies.

This study proposes a protocol to obtain a representation of the epigenetic landscape of a gene regulatory network described by a continuous dynamical system of protein concentrations. Specifically, the research aims to elucidate the epigenetic landscape associated with *Arabidopsis thaliana*’s flower morphogenesis process. The genetic regulation network that describes this process is already known, comprising 12 nodes representing the genes involved in the process, and the discrete dynamics describing their relationships are already well studied [37, 38].

**Fig. 1:**
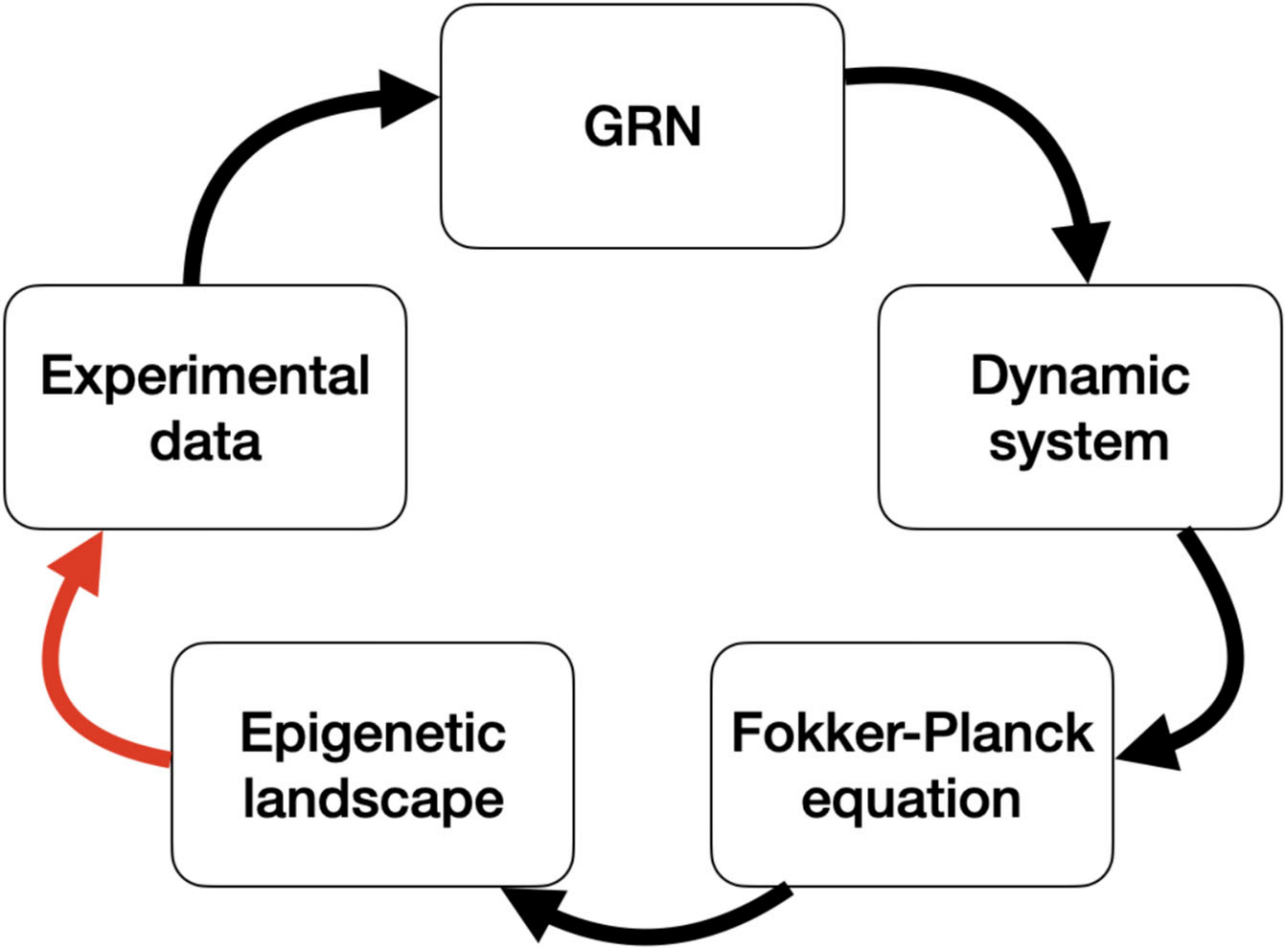
Generally, the study of cell development processes starts from the experimental data available to create a GRN. Subsequently, a GRN is proposed based on data. A dynamic system can be proposed based on the GRN. A FPE can be derived from the dynamic system. The epigenetic landscape can be associated with the solution of FPE. The epigenetic landscape can be used to find simulated experimental data from the model. This data can be used to validate or discriminate the model

A continuous time model for the *Arabidopsis thaliana*’s flower morphogenesis is proposed to generalize the discrete dynamics presented in [37, 38]. The Fokker-Planck equation (FPE) associated with the model is constructed, and a gamma mixture model is utilized to estimate its stationary solution. The stationary solution is subsequently employed to evaluate correlations in the genetic expressions that should be observed in an experimental setting [38, 39]. The free energy related to the stationary solution of the FPE is identified as the epigenetic landscape. This methodology will facilitate establishing a clear relationship between experimental data and theory, enabling the discrimination of different model proposals and thus allowing for the inference of a theoretical model from experimental results (see Fig. 1). Moreover, it would constitute a significant advancement towards inferring the phenotypic consequences resulting from changes in the genotype through theoretical means.

## Methods

### Gene Regulatory Network

**Fig. 2:**
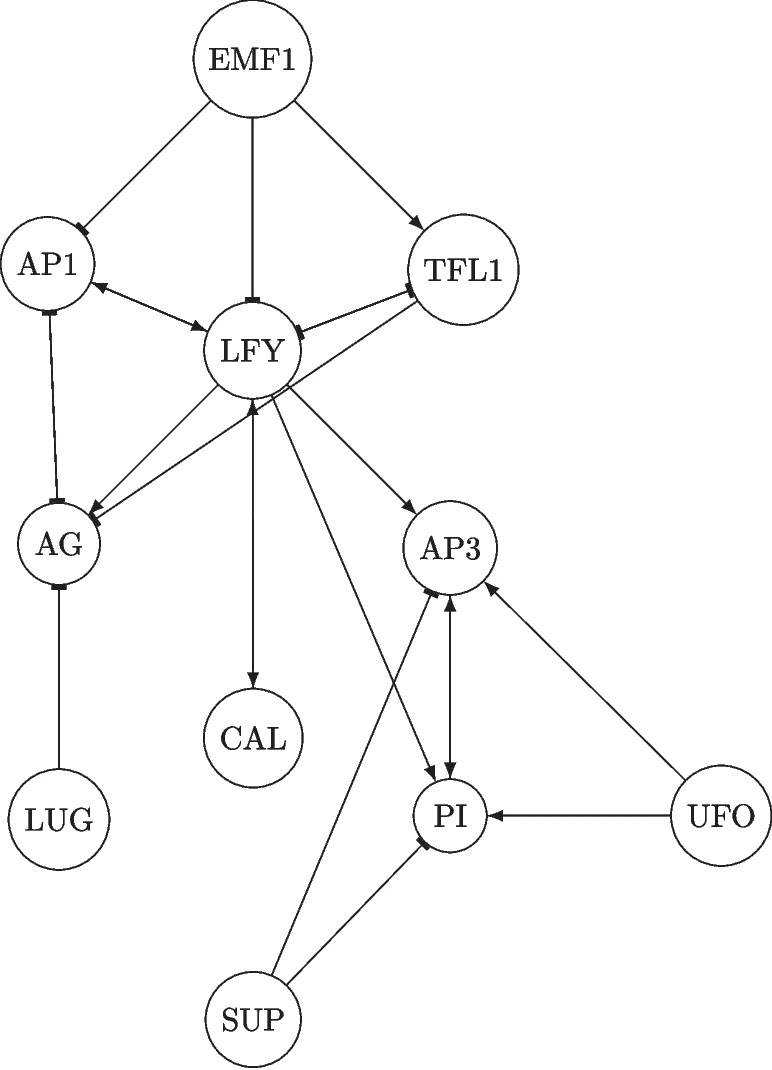
Gene regulatory network of *Arabidopsis thaliana* morphogenesis

Here, we will work with a well-studied GRN for which experimental data on gene coexpression are available. The data will allow us to test the building process of the model and its solution procedure. We construct a minimal continues model for the *Arabidopsis thaliana*’s flower morphogenesis from minimal information of the interaction between involved genes. The initial information is if a particular gene promotes or inhibits the activation of other genes, but a quantitative measure of the intensity of this interaction is unknown. A GRN that describes the qualitative relation between genes involved in AT flower morphology is proposed in [37]. In this network, each node represents a gene that takes part in such a developmental process. The name and order of those genes are shown in Table 1, and the network is shown in Fig. 2. With this limited information, proposing a Boolean model [40] is the first step to translating these gene relations into a GRN model. Mendoza and Állvarez Buylla presented a Boolean model representing the temporal evolution of this system, which is given by

1 $$\begin{aligned} x_i(t+1)=\textbf{H}\left( \sum _{j=1}^N w_{ij} x_j(t)-\theta _{i}\right) , \end{aligned}$$

where $\textbf{H}$ is a step function defined by

2 $$\begin{aligned} \textbf{H}(x)= {\left\{ \begin{array}{ll} 1&  \quad \text {if} \quad x>0\\ 0&  \quad \text {if} \quad x\le 0\end{array}\right. } \end{aligned}$$

where $w_{ij}$ equals the intensity of the interaction of the j-th over the i-th gene.

Those gene states can be active or inactive and are represented by values 1 and 0, respectively. So $x_i = 1$ if the weighted sum of all genes that regulate it exceeds a threshold value $\theta _i$.

**Table 1:** List of the genes involved in the *Arabidopsis thaliana* morphogenesis

1	EMF1	3	LFY	5	CAL	7	UFO	9	AG	11	PI
2	TFL1	4	AP1	6	LUG	8	BFU	10	AP3	12	SUP

In addition, in [37], a genetic search algorithm [41] was used to find integer values for $w_{ij}$ and $\theta _{i}$ that lead to at least four stationary states corresponding to 4 phenotypic stages in the developmental process of the *Arabidopsis thaliana*’s flower [42]. The weight matrix *W* and the threshold vector $\theta$, whose entries correspond to the parameters $w_{ij}$ and $\theta _i$, were found to be

$$\begin{aligned} W=\left[ \begin{array}{cccccccccccc} 0 &  0 &  0 &  0 &  0 &  0 &  0 &  0 &  0 &  0 &  0 &  0 \\ 1 &  0 &  -2 &  0 &  0 &  0 &  0 &  0 &  0 &  0 &  0 &  0 \\ -2 &  -1 &  0 &  2 &  1 &  0 &  0 &  0 &  0 &  0 &  0 &  0 \\ -1 &  0 &  5 &  0 &  0 &  0 &  0 &  0 &  -1 &  0 &  0 &  0 \\ 0 &  0 &  2 &  0 &  0 &  0 &  0 &  0 &  0 &  0 &  0 &  0 \\ 0 &  0 &  0 &  0 &  0 &  0 &  0 &  0 &  0 &  0 &  0 &  0 \\ 0 &  0 &  0 &  0 &  0 &  0 &  0 &  0 &  0 &  0 &  0 &  0 \\ 0 &  0 &  0 &  0 &  0 &  0 &  0 &  0 &  0 &  1 &  1 &  0 \\ 0 &  -2 &  1 &  -2 &  0 &  -1 &  0 &  0 &  0 &  0 &  0 &  0 \\ 0 &  0 &  3 &  0 &  0 &  0 &  2 &  1 &  0 &  0 &  0 &  -2 \\ 0 &  0 &  4 &  0 &  0 &  0 &  1 &  1 &  0 &  0 &  0 &  -1 \\ 0 &  0 &  0 &  0 &  0 &  0 &  0 &  0 &  0 &  0 &  0 &  0 \end{array}\right] \quad \theta = \begin{bmatrix} 0 \\ 0 \\ 3 \\ -1 \\ 1 \\ 0 \\ 0 \\ 1 \\ -1 \\ 0 \\ 0 \\ 0 \end{bmatrix} \end{aligned}$$

In this matrix, the negative signs indicate the repression of the i-th row gene due to the j-th column gene.

### Continuous model

From the discrete time GRN dynamics (1), a continuous time model describing the protein concentrations involved in the morphogenesis of the AT flower was built. The model is based on a system of ordinary differential equations describing the evolution of the protein concentrations involved in the developmental process.

Each node of the GRN represents a gene, and we assumed that each gene $G_{a}$ is activated by protein $P_{b}$, then it transcribes mRNA, and in turn, a corresponding protein $P_{a}$ is translated. That is to say, for each one of the 12 nodes from the GNR, two differential equations are proposed. One of these equations will describe the temporal evolution of the protein concentration associated with each gene, and the other the transcribed mRNA.

The reaction scheme assumed for transcription of mRNA is given by

$$\begin{aligned} G_{a} + n_{ab}P_b \xrightarrow {k_{ab}^{+}} G_{a}^{*} ,&\qquad G_{a}^{*} \xrightarrow {k_{ab}^{-}} G_{a} + n_{ab}P_{b}\\ G_{a}^{*} \xrightarrow {\alpha _{ab}} G_{a}^{*} + mRNA_{a} ,&\qquad mRNA_{a} \xrightarrow {\gamma _{a}} \emptyset \\ mRNA_{a} \xrightarrow {\beta _{a}} mRNA_{a} + P_{a} ,&\qquad P_{a}\xrightarrow {\delta _{a}}\emptyset \end{aligned}$$

The first two reactions are faster than the others, then active gene $G_{a}^{*}$ concentration can be approximated at its quasi-steady state value. Then, the differential equations for the mRNA concentration $m_{a}$ and protein $P_{a}$ concentration are

3 $$\begin{aligned} \frac{dm_a}{dt}=\frac{\alpha _{ab}k_{ab}p_b^{n_{ab}}}{1+k_{ab}p_b^{n_{ab}}}-\gamma _am_a \end{aligned}$$

4 $$\begin{aligned} \frac{dp_a}{dt}=\beta _am_a-\delta _ap_a \end{aligned}$$

where $k_{ab}=k_{ab}^{+}/k_{ab}^{-}$. These equations describe the activation of the protein (*A*) production by means of the corresponding mRNA by the concentration of protein *B*.

On the other hand, the basic reaction scheme of a repressor is

$$\begin{aligned} G_{a} + n_{ab}P_b \xrightarrow {k_{ab}^{+}} G_{a}^{*} ,&\qquad G_{a}^{*} \xrightarrow {k_{ab}^{-}} G_{a} + n_{ab}P_{b}\\ G_{a} \xrightarrow {\alpha _{ab}} G_{a} + mRNA_{a} ,&\qquad mRNA_{a} \xrightarrow {\gamma _{a}} \emptyset \\ mRNA_{a} \xrightarrow {\beta _{a}} mRNA_{a} + P_{a} ,&\qquad P_{a}\xrightarrow {\delta _{a}}\emptyset \end{aligned}$$

At the quasi-steady state of the first two reactions, the corresponding differential equations describing the protein $P_{a}$ and *mRNA* concentrations are

5 $$\begin{aligned} \frac{dm_a}{dt}=\frac{\alpha _{ab}}{1+k_{ab}p_b^{n_{ab}}}-\gamma _am_a \end{aligned}$$

6 $$\begin{aligned} \frac{dp_a}{dt}=\beta _am_a-\delta _ap_a \end{aligned}$$

These equations describe the repression of a protein (*A*) production via the corresponding mRNA by a protein *B*.

In these equations, $p_a$ is the concentration of protein *A*, $m_a$ is the concentration of mRNA associated with protein *A*, $p_b$ is the concentration of protein *B*, which regulates protein *A* production, and $m_b$ is the concentration of mRNA associated with protein *B*. Parameters $\alpha _{ab}$, $k_{ab}$, and $n_{ab}$ describe the way protein *B* concentration alters protein *A* concentration, while $\gamma _{a}$ and $\delta _a$ are the mRNA and protein degradation rate, respectively.

This approach would yield two equations for each node in the GRN: one for the protein and one for the corresponding mRNA. However, to reduce the system’s dimensionality, an approximation is made based on the difference between the time scales of both processes. Specifically, mRNA production rapidly reaches its stationary state while protein production continues to evolve. Namely, we assume $\frac{dm}{dt}=0$ and so

7 $$\begin{aligned} 0=\frac{\alpha _{ab}k_{ab}p_b^{n_{ab}}}{1+k_{ab}p_b^{n_{ab}}}+\alpha _{a0}-\gamma _am_a \end{aligned}$$

Therefore,

8 $$\begin{aligned} \Rightarrow m_a=\frac{\alpha _{ab}k_{ab}p_b^{n_{ab}}}{\gamma _a(1+k_{ab}p_b^{n_{ab}})}+\frac{\alpha _{a0}}{\gamma _a} \end{aligned}$$

Substituting in the equation for the protein production,

9 $$\begin{aligned} \frac{dp_a}{dt}=\frac{\beta _a}{\gamma _a}\frac{\alpha _{ab}k_{ab}p_b^{n_{ab}}}{1+k_{ab}p_b^{n_{ab}}}+\frac{\beta _a\alpha _{a0}}{\gamma _a}-\delta _ap_a \end{aligned}$$

Similarly, for the protein repression equation,

10 $$\begin{aligned} \frac{dp_a}{dt}=\frac{\beta _a}{\gamma _a}\frac{\alpha _{ab}}{1+k_{ab}p_b^{n_{ab}}}+\frac{\beta _a\alpha _{a0}}{\gamma _a}-\delta _a p_a \end{aligned}$$

We now have an equation for each one of the 12 proteins involved in the morphogenesis process.

We now need to relate the Boolean model parameters $w_{ij}$ and $\theta _{i}$ to the parameters of the reaction schemes for a repressor. $w_{ij}$ corresponds to the sensitivity to activation or repression of gene *i* from another gene *j*. It is natural to associate it with the activation rate $k_{ij}^{+}$. If $w_{ij}$ is positive, then it indicates that *j* activates *i*, and if $w_{ij}$ is negative, then *j* represses *i*. On the other hand, $\theta$ is described as the threshold value of the interaction from other genes to be activated. Because the activation rate must be greater than the inactivation rate of the gene to reach its activation state, it is possible to relate the absolute value of theta with the inactivation rate $k_{ij}^{-}$ and its sign indicating if gene *i* has a basal synthesis rate corresponding for negative $\theta$ or if positive, there is not basal synthesis rate. As there are some values of $\theta$ equal to 0 and the deactivation rate should be greater than 0, we assume the deactivation rate has a basal value plus the absolute value of $\theta$. Due to the lack of more information in the Boolean model and to avoid introducing fictitious information, we choose a parsimonious model, setting all other parameters to 1.

Another issue that must be addressed is that equations (9) and (10) describe a protein *B* modifying the production of a protein *A*; however, the production of a protein may depend on more than one protein. Thus, we must use a more general Hill function that considers the joint action of multiple proteins.

Thus, we employ the Hill functions

11 $$\begin{aligned} \frac{dp_i}{dt}=\frac{a}{a +\displaystyle \sum _{j}k_{i,j}p_j^n} \end{aligned}$$

and

12 $$\begin{aligned} \frac{dp_i}{dt}=\frac{\displaystyle \sum _{j}k_{i,j}p_j^n}{a +\displaystyle \sum _{j}k_{i,j}p_j^n} \end{aligned}$$

for the protein concentration repression and activation, respectively [43].

On the other hand, the *a* parameters, called the *activation coefficients*, can be interpreted as the j-th protein concentration needed to activate the i-th protein production; therefore, they are analogous to $\theta$ parameters derived in [37].

Based on the above, we propose an ODEs system representing the continuous dynamics of the proteins concentration involved in the morphogenesis of the AT flower shown in (13).

13 $$\begin{aligned} \frac{dp_1}{dt}= &   \alpha _{1,0}-\delta _1 p_1 \nonumber \\ \frac{dp_2}{dt}= &   \frac{1+|\theta _2|+|w_{2,1}|p_1}{1+|\theta _2|+|w_{2,1}|p_1+|w_{2,3}|p_3}+\alpha _{2,0}-\delta _2 p_2 \nonumber \\ \frac{dp_3}{dt}= &   \frac{|w_{3,4}|p_4+|w_{3,5}|p_5}{1+|\theta _3|+|w_{3,1}|p_1+|w_{3,2}|p_2+|w_{3,4}|p_4+|w_{3,5}|p_5}+\alpha _{3,0}-\delta _3 p_3 \nonumber \\ \frac{dp_4}{dt}= &   \frac{1+|\theta _4|+w_{4,3}|p_3}{1+|\theta _4|+|w_{4,1}|p_1+|w_{4,3}|p_3+|w_{4,9}|p_9}+\alpha _{4,0}-\delta _4 p_4 \nonumber \\ \frac{dp_5}{dt}= &   \frac{|w_{5,3}|p_3}{1+|\theta _5|+|w_{5,3}|p_3}+\alpha _{5,0}-\delta _5 p_5 \nonumber \\ \frac{dp_6}{dt}= &   \alpha _{6,0}-\delta _6 p_6 \nonumber \\ \frac{dp_7}{dt}= &   \alpha _{7,0}-\delta _7 p_7 \nonumber \\ \frac{dp_8}{dt}= &   \frac{|w_{8,10}|p_{10}+|w_{8,11}|p_{11}}{1+|\theta _8|+|w_{8,10}|p_{10}+|w_{8,11}|p_{11}}+\alpha _{8,0}-\delta _8 p_8 \nonumber \\ \frac{dp_9}{dt}= &   \frac{1+|\theta _9|+|w_{9,3}|p_{3}}{1+|\theta _9|+|w_{9,2}|p_{2}+|w_{9,3}|p_{3}+|w_{9,4}|p_{4}+|w_{9,6}|p_{6}}+\alpha _{9,0}-\delta _9 p_9 \nonumber \\ \frac{dp_{10}}{dt}= &   \frac{1+|\theta _{10}|+|w_{10,3}|p_{3}+|w_{10,7}|p_{7}+|w_{10,8}|p_{8}}{1+|\theta _{10}|+|w_{10,3}|p_{3}+|w_{10,7}|p_{7}+|w_{10,8}|p_{8}+|w_{10,12}|p_{12}}+\alpha _{10,0}-\delta _{10} p_{10} \nonumber \\ \frac{dp_{11}}{dt}= &   \frac{1+|\theta _{11}|+|w_{11,3}|p_{3}+|w_{11,7}|p_{7}+|w_{11,8}|p_{8}}{1+|\theta _{11}|+|w_{11,3}|p_{3}+|w_{11,7}|p_{7}+|w_{11,8}|p_{8}+|w_{11,12}|p_{12}}+\alpha _{11,0}-\delta _{11} p_{11} \nonumber \\ \frac{dp_{12}}{dt}= &   \alpha _{12,0}-\delta _{12} p_{12} \end{aligned}$$

### Experimental data

*Arabidopsis thaliana* serves as a model organism, and experimental data regarding its processes are abundant and readily available. In particular, the work of Obayashi et al. [44] provides access to condition-independent coexpression data derived from publicly available RNA-seq and microarray data.

Each microarray analysis offers information about coexpression levels for thousands of genes simultaneously. Projects as in [44] contain large collections of microarray data, which allows us to access information about changes in transcript levels in these datasets even when such microarray was not intended for the same purpose.

It is possible to search at the project’s site, coexpression data for each pair of genes of interest, and then calculate the Pearson correlation coefficient between them, given by

$$\begin{aligned} \rho =\frac{\sigma _{xy}}{\sigma _x\sigma _y} \end{aligned}$$

**Fig. 3:**
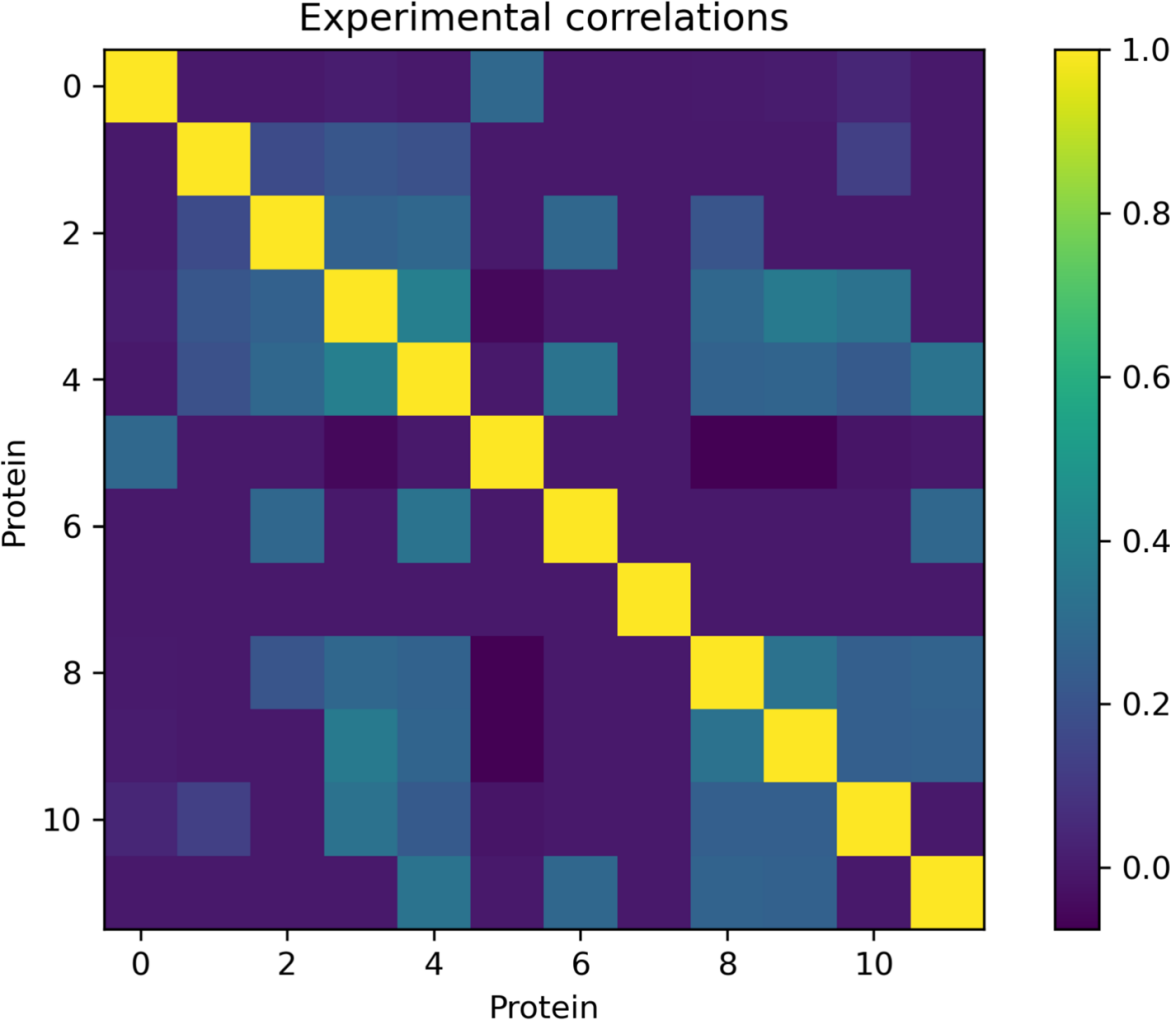
Correlation matrix obtained from experimental data. Missing data is set to zero

This was carried out for each pair of genes involved in the process of interest in Table 1. These correlation values were arranged in matrix $M_e$, as illustrated in Fig. 3, and will be compared with the results obtained from the theoretical model.

### Solution to the Fokker-Planck equation

The variations in measurements of coexpression levels result from the system being influenced by many other variables and factors not explicitly taken into account. Thus, we must find a solution to the associated stochastic model with extrinsic fluctuations to obtain inferred correlations between protein concentrations from the theoretical model. Furthermore, stationarity can be assumed based on the experimental data.

Recall the multivariate chemical Langevin equation [45]

14 $$\begin{aligned} dp_i=A_{i}(\textbf{p},t)dt+\sum _j^dc_{ij}(\textbf{p},t)\eta _j(t)dt \end{aligned}$$

that describes the changes in the system states through time with fluctuations. For system, (13) can write $A_{i}(\textbf{p},t)=B_{i}(\textbf{p},t) - \delta _{i} p_{i}$, where $B_{i}$ represent the positive part of the equations. Also, if $\eta _{j}$ are all independent identical Gaussian white noise processes, then $\sum _j^dc_{ij}(\textbf{p},t)\eta _j(t) = \frac{1}{\sqrt{\Omega }}(\sqrt{B_{i}(\textbf{p},t) + \delta _{i}p_{i}})\eta$. Here, $\eta$ is a Gaussian white noise process identical to $\eta _{i}$, and $\Omega$ is the system size [46–48]. Thus, the chemical Langevin equation for the system (13) can be expressed as

15 $$\begin{aligned} dp_{i} = (B_{i}(\textbf{p},t) - \delta _{i} p_{i})dt + \frac{1}{\sqrt{\Omega }}(\sqrt{B_{i}(\textbf{p},t) + \delta _{i}p_{i}})\eta dt \end{aligned}$$

This set of equations is equivalent to the Fokker-Planck equation (FPE) [45, 49]

16 $$\begin{aligned} \frac{\partial P}{\partial t}=-\sum _{j}\frac{\partial }{\partial p_j}\left[ \left( B_{j}(\textbf{p},t) - \delta _{j} p_{j}\right) P\right] +\frac{1}{2}\sum _{j,k}\frac{\partial ^2}{\partial p_j\partial p_k}(\Gamma _{j}(\textbf{p},t) P) \end{aligned}$$

where $\Gamma _{j}(\textbf{p},t)=\frac{1}{\Omega }(B_{i}(\textbf{p},t) + \delta _{i}p_{i})$. It is worth noting that we have used the Ito interpretation of integration to go from (15) to (16); thus, in this representation, the drift term is not affected by the noise term $\Gamma _{j}$.

To find the epigenetic landscape of the system of interest, we solve the multivariate FPE (16) associated with the dynamics given by the proposed ODE system. This equation describes the probability density function of temporal evolution for our system. So, by solving it, we obtain the probability value *P* for each of the points in the state space of the system $\textbf{y}$, after a given time *t*. This equation is related to the epigenetic landscape since, in its stationary version, the local maxima of the solution, which are the most probable states, correspond to the attractors in the energy potential and vice versa.

In the same spirit of previous results about the type of distributions a GRN presents [35, 50], we propose a gamma mixture model as the solution of the stationary FPE

17 $$\begin{aligned} \hat{\mathcal {P}}=\sum _{i=1}^{n}A_i P_{i} \end{aligned}$$

with

$$\begin{aligned} P_{i} = \prod _{j}^{12} \frac{\beta _{ij}^{\alpha _{ij}}}{\Gamma (\alpha _{ij})^{\alpha _{i}}}p_{j}^{\alpha _{ij}-1}\exp {(-\beta _{ij}p_{j})}. \end{aligned}$$

Here, *n* is the number of stationary states, the $\alpha _{ij}$’s and $\beta _{ij}$’s are the shapes and rates of each gamma distribution, and $p_{j}'s$ are the concentrations of the different proteins.

Expression (17) is an ansatz for the solution to the stationary FPE

18 $$\begin{aligned} 0=-\sum _{j}\frac{\partial }{\partial p_j}\left[ \left( B_{j}(\textbf{p},t) - \delta _{j} p_{j}\right) P\right] +\frac{1}{2}\sum _{j,k}\frac{\partial ^2}{\partial p_j\partial p_k}(\Gamma _{j}(\textbf{p},t) P) \end{aligned}$$

**Table 2:** Parameters for the gamma mixure model, location of the four attractors, and mean concentration of the proteins of activated genes in each attractor

Parameter		Value
$\alpha _{1j}$ (shape)	=	(1, 2.25, 2.22, 1, 2.23, 2.24, 2.24, 2.23, 2.24, 2.24, 2.26, 1 )
$\beta _{1j}$ (rate)	=	(0.91, 0.64, 0.64, 0.89, 0.64, 0.64, 0.64, 0.64, 0.64, 0.64, 0.64, 0.91)
Mode $(\alpha - 1)/\beta$		
(location)	=	(0, 1.93, 1.88, 0, 1.89, 1.91, 1.92, 1.90, 1.92, 1.91, 1.94, 0 )
Mean conc		
(active genes)	=	1.9
$\alpha _{2j}$ (shape)	=	(5.83, 5.79, 1, 5.82, 5.82, 5.74, 1, 5.66, 1, 5.69, 1, 5.78 )
$\beta _{2j}$ (rate)	=	(1.88, 1.87, 0.95, 1.86, 1.87, 1.86, 0.93, 1.85, 0.94, 1.85, 0.94, 1.87)
Mode $(\alpha - 1)/\beta$		
(location)	=	(2.56, 2.55, 0, 2.58, 2.57, 2.54, 0, 2.51, 0, 2.52, 0, 2.55)
Mean conc		
(active genes)	=	2.5
$\alpha _{3j}$ (shape)	=	(1, 1, 2.21, 2.21, 2.22, 2.22, 2.21, 1, 2.21, 1, 2.22, 2.22 )
$\beta _{3j}$ (rate)	=	(0.92, 0.93, 0.67, 0.67, 0.67, 0.67, 0.67, 0.94, 0.67, 0.94, 0.66, 0.67 )
Mode $(\alpha - 1)/\beta$		
(location)	=	(0, 0, 1.79, 1.79, 1.82, 1.81, 1.81, 0, 1.79, 0, 1.83, 1.81)
Mean conc		
(active genes)	=	1.8
$\alpha _{4j}$ (shape)	=	(2.21, 1, 2.21, 2.23, 1, 1, 1, 2.19, 2.24, 2.24, 1, 2.22)
$\beta _{4j}$ (rate)	=	(0.63, 0.89, 0.63, 0.63, 0.9, 0.92, 0.89, 0.64, 0.63, 0.63, 0.89, 0.63 )
Mode $(\alpha - 1)/\beta$		
(location)	=	(1.91, 0, 1.91, 1.94, 0, 0, 0, 1.86, 1.96, 1.95, 0 1.91)
Mean conc		
(active genes)	=	1.9

We use the least square weighted residual method [51] to find the parameters *n*, $A_i$, $y_{j,0}$, and $\sigma _{i,j}$ that solve equation (18); in this way, the problem of solving the stationary FPE becomes an optimization problem. That is, we seek to minimize

19 $$\begin{aligned} \int _{D}\Vert R(\textbf{p})\Vert dD \end{aligned}$$

where $R(\textbf{p})$ is the residual in the equation

20 $$\begin{aligned} R(\textbf{p})=-\sum _{j}\frac{\partial }{\partial p_j}\left[ \left( B_{j}(\textbf{p},t) - \delta _{j} p_{j}\right) \hat{P}\right] +\frac{1}{2}\sum _{j,k}\frac{\partial ^2}{\partial p_j\partial p_k}(\Gamma _{j}(\textbf{p},t) \hat{P}). \end{aligned}$$

The integral (19) is solved using Monte Carlo quadrature/collocation [52] with $N=10^{6}$ collocation points. The quadrature is computed each time a new set of parameters requires its evaluation by the algorithm that seeks its minimization.

**Fig. 4:**
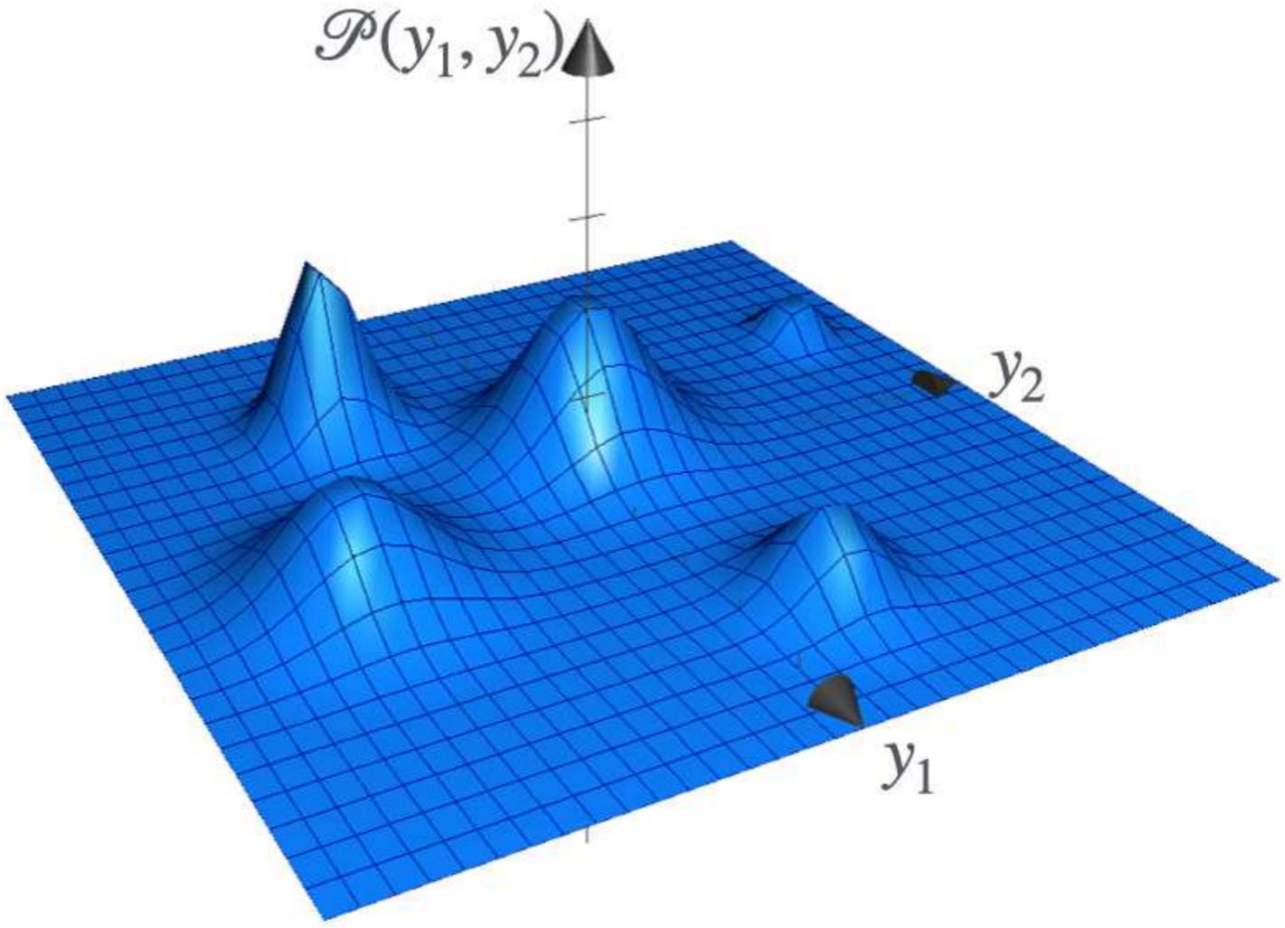
Graph of the function (17) when $d=2$ and $n=5$. The maxima correspond to the stationary states of the system. For the system of interest, *d* has to be equal to 12

Due to the relatively slow decay of the gamma distribution’s tails, calculating the integral (19) can lead to wide variations across samples. This occurs because fluctuations in the sampling points can be considerable within a 12-dimensional space. To mitigate this problem, a Gaussian mixture model was initially used as a proxy distribution. This distribution helps identify regions close to the dynamical system’s attractors and the variances of the fluctuations around them (Fig. 4). The centers and variances of the normal distributions within the Gaussian mixture model can then be used as a starting point for fine-tuning the gamma mixture model. The model fitting was performed in three stages: (1) a broad parameter space search using Monte Carlo methods with a Gaussian mixture model to locate the attractor regions and their variances; (2) a refined search for the position and variance of the system’s attractor regions using gradient descent-based minimization methods; and (3) the position and variance found in the previous step were used to initialize the parameters of the Gamma mixture model, followed by fine-tuning using gradient descent-based minimization algorithms. Each stage is detailed further below.

In the first stage, we employ the tree-structured parsen estimator (TPE) algorithm [53] to address the optimization problem. TPE is a variant of the Bayesian method [54, 55] that has demonstrated notable success in hyperparameter optimization [56, 57]. For this purpose, we utilized the package Optuna 4.1.0 in Python 3.12. In the subsequent stage, the Adam optimization algorithm [58] was employed to find the parameters of the Gaussian mixture model (MGM) that minimize the residual (19). The Tensorflow 2.18 and Tensorflow Probability 0.25 libraries in Python 3.11 were utilized to leverage automatic differentiation capabilities [59]. In this stage and the following one, the Tensorflow Probability library was used to represent both the Gaussian mixture distribution and the gamma mixture distribution for the next stage. Finally, the last stage commenced with a gamma mixture, initializing the modes of the mixture distribution with the locations from the Gaussian model and adjusting the variances of the gamma distributions based on the variances of the Gaussian mixture model. Subsequently, the Adam optimization algorithm was employed to fine-tune the gamma mixture. The algorithm was stopped when no significant decrease in the residual was observed, achieving an average value per collocation point of $10^{-6}$. The resulting values and the respective locations of the attractors are presented in Table 2.

Upon estimation of the FPE solution *P*, it becomes feasible to identify the epigenetic landscape as $U(y)=-\ln {P(y)}$.

We can now calculate the correlations between pairs of proteins and compare them with experimental observations. It is worth noticing that, in this case, we are not able to use traditional hypothesis tests like (Kolmogorov-Smirnov, Wald-Wolfowitz) for a single variable, neither multivariate generalizations [60–63] because experimental data is normally reported as coexpression matrices rather than concentration samples.

We take a sample of $10^{5}$ points from the estimated $\hat{\mathcal {P}}$ distribution. From this sample, the Pearson correlation was calculated for each pair of variables, and a matrix of correlations $M_m$ may be derived. This matrix can be compared directly with the matrix $M_{e}$ shown in Fig. 3 obtained from gene expression measures for model validation and comparison.

A straightforward comparison is achieved by calculating the Euclidean distance between both matrices, that is,

21 $$\begin{aligned} d(M,N)=\sqrt{\sum _{s=1}^{t^2}(m_{s}-n_{s})^2} \end{aligned}$$

where *M* and *N* are matrices both with dimension $t\times t$ and $m_s$ and $n_s$ are their respective entries.

## Results

**Fig. 5:**
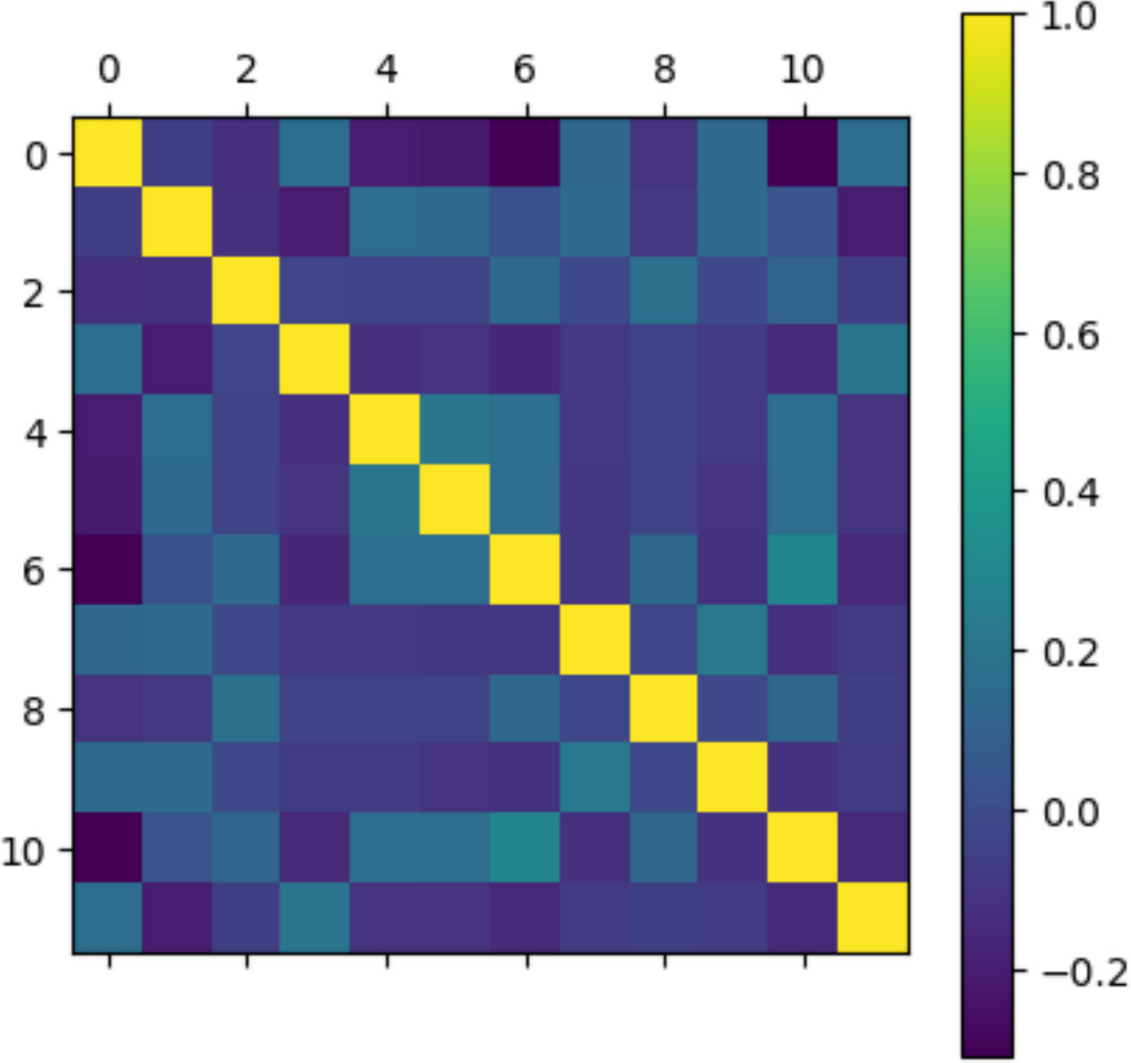
Deduced correlations from the gene regulatory network. Numbers correspond to gene labels

The theoretical correlation matrix $M_m$ that was deduced from the solution to the FPE $\hat{\mathcal {P}}$ is represented in Fig. 5.

Then, the Euclidean distance

22 $$\begin{aligned} D=d(M_e,M_m)=2.68 \end{aligned}$$

was measured between the experimental data matrix and the theoretical correlations matrix obtained from the model. To make this value meaningful, we must calculate how significant this distance is compared to an uninformative correlation matrix.

To do so, two sets of distances were derived. The first one is taken from samples of the distances between two random matrices with the same characteristics of a correlation matrix; the second one is taken from a random sample of distances between the experimental matrix $M_e$ and random matrices of correlations.

**Fig. 6:**
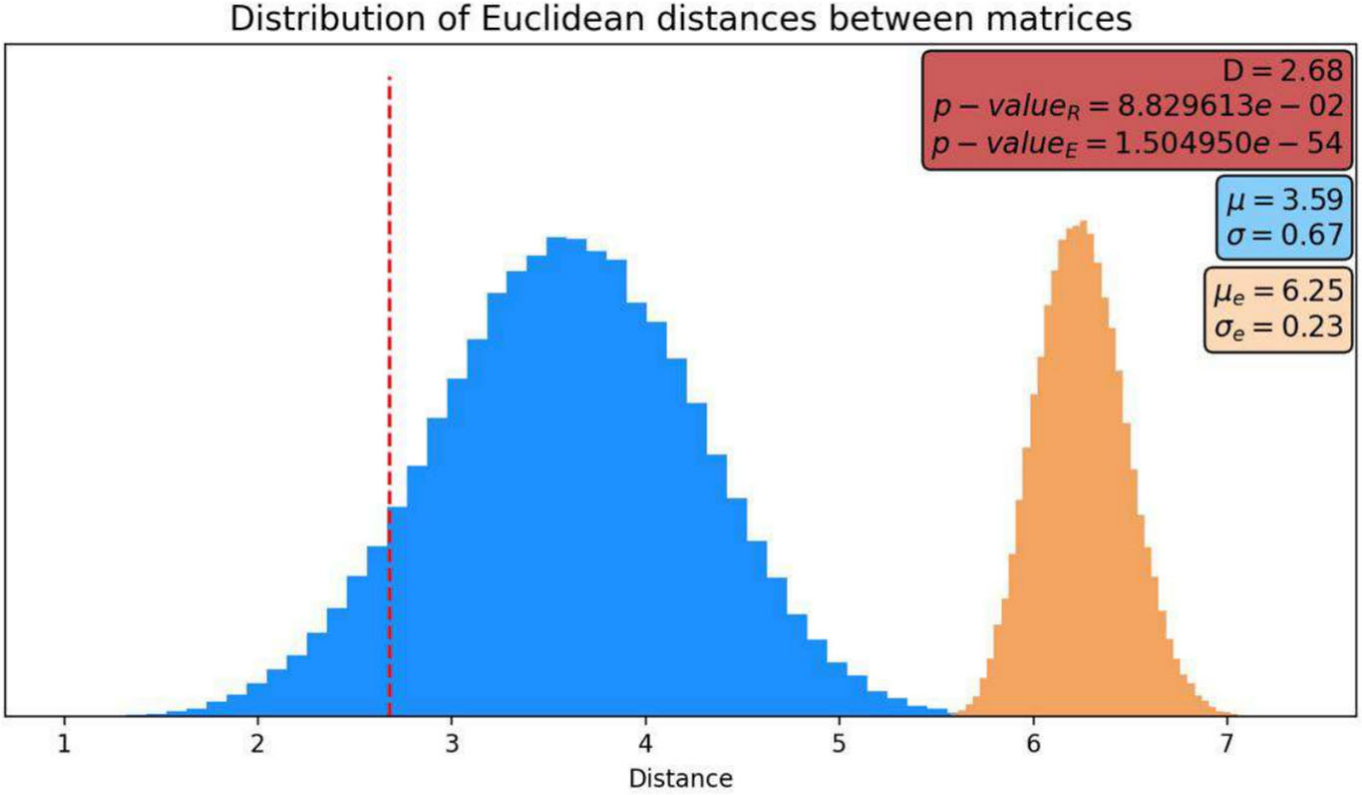
Distance between the model correlation matrix and the experimental data matrix. The orange histogram shows the distribution of the distances between the experimental data matrix and a random matrix of the same characteristics. The blue histogram shows the distributions of the distances between two random matrices of the same characteristics as the experimental data matrix

This allows us to infer the corresponding distance distributions shown in Fig. 6. The distance $d(M_e,M_m)$ was compared with both distributions, showing that up to this test, the model cannot be discarded.

The epigenetic landscape can provide relevant information regarding cellular processes. Specifically, the depth of each potential well offers insights into escape probability and mean escape time. The distance between wells yields information concerning the transition probability between distinct attractors. Thus, the most probable transitions between attractors and, in particular, cell processes could be determined, as well as the relative time spent in each cell state.

## Discussion

Adequate knowledge of the epigenetic landscape is a relevant problem in systems biology and epigenetics. Various approaches have been proposed and implemented [64–67]. In this study, we propose a practical methodology for estimating it for systems with many dimensions. This method involves deriving the Fokker-Planck equation (FPE) that describes the dynamics of a cellular process from previous knowledge of its GRN. The epigenetic landscape *U*(*y*) is associated with the stationary solution $P_{ss}(y)$ of the FPE as $U(y)=-\ln {P_{ss}(y)}$ [66]. A gamma mixture model is proposed as a solution for $P_{ss}$ where the search for relevant parameters takes place in three stages. In the first stage, a global exploration is conducted using a Monte Carlo algorithm to search for the attractor regions. In the second stage, a Gaussian mixture model is employed as a proxy distribution to estimate the location of the attractors and the variance of the dynamic system around them. The Gaussian mixture is used because its quadrature points are not widely spread out, and the fluctuations in each evaluation are small, unlike with a gamma distribution. Finally, the location and variance found are used to initialize the parameter search for the gamma mixture model. Gradient-based optimization algorithms utilizing automatic differentiation are employed in the last two stages. The FPE solution can be used to validate the underlying model using experimental co-expression data. A Monte Carlo-Hastings algorithm was employed to sample from the derived distribution, and correlations between protein concentrations were directly compared with correlations observed in the experimental co-expression data. Similarly, the solution of the FPE can be used to infer missing gene expression data from existing experimental data. To illustrate this procedure, we found the epigenetic landscape of the FPE for the *Arabidopsis thaliana* flower morphogenesis process. Furthermore, the estimation of the relative Manhattan distances between potential wells of the epigenetic landscape can be utilized to determine the most probable transition between cell states, and their relative heights can provide the relative duration spent in each attractor. For the *Arabidopsis thaliana* regulatory network analyzed herein, this duration is correlated with the time spent in each developmental phase. It is worth noting that gene regulatory networks inferred from Boolean models are inherently limited in the information they capture, a consequence of the models’ simplicity. Therefore, these models are not meant to provide a detailed description of a GRN; rather, their aim is to highlight general relationships among protein concentrations across the network’s distinct attractors. On the other hand, it should be noted that mRNA and protein production are bursty, and therefore, the present approach using the Langevin chemical equation based on white noise, for such complex processes like mRNA and protein production, is an approximation. It has recently been observed that stochastic models based on compound Poisson processes provide a good description of mRNA and protein expression levels [68, 69]. A continuous approximation of the chemical master equation could be employed in the future, along with the techniques described here, to find more reliable probability distributions for protein concentrations.

## Data Availability

The dataset of gene coexpression for *Arabidopsis thaliana* is available at https://doi.org/10.1093/pcp/pcac041 or https://atted.jp/.
